# CGMD Platform: Integrated Web Servers for the Preparation, Running, and Analysis of Coarse-Grained Molecular Dynamics Simulations

**DOI:** 10.3390/molecules25245934

**Published:** 2020-12-15

**Authors:** Alessandro Marchetto, Zeineb Si Chaib, Carlo Alberto Rossi, Rui Ribeiro, Sergio Pantano, Giulia Rossetti, Alejandro Giorgetti

**Affiliations:** 1 Department of Biotechnology, University of Verona, 37134 Verona, Italy; a.marchetto@fz-juelich.de (A.M.); carloalberto.rossi@studenti.univr.it (C.A.R.); ruipedro.fernandesribeiro@univr.it (R.R.); 2 Computational Biomedicine, Institute for Neuroscience and Medicine (INM-9) and Institute for Advanced Simulations (IAS-5), Forschungszentrum Jülich, 52425 Jülich, Germany; z.si.chaib@fz-juelich.de; 3 Faculty of Mathematics, Computer Science and Natural Sciences, RWTH Aachen University, 52062 Aachen, Germany; 4 Institut Pasteur de Montevideo, Mataojo 2020, 11400 Montevideo, Uruguay; spantano@pasteur.edu.uy; 5 Jülich Supercomputing Center (JSC), Forschungszentrum Jülich, 52428 Jülich, Germany; 6 Department of Hematology, Oncology, Hemostaseology, and Stem Cell Transplantation, RWTH Aachen University, 52062 Aachen, Germany

**Keywords:** CGMD, SIRAH, martini force field, backmapping

## Abstract

Advances in coarse-grained molecular dynamics (CGMD) simulations have extended the use of computational studies on biological macromolecules and their complexes, as well as the interactions of membrane protein and lipid complexes at a reduced level of representation, allowing longer and larger molecular dynamics simulations. Here, we present a computational platform dedicated to the preparation, running, and analysis of CGMD simulations. The platform is built on a completely revisited version of our *Martini coarsE gRained MembrAne proteIn Dynamics* (MERMAID) web server, and it integrates this with other three dedicated services. In its current version, the platform expands the existing implementation of the Martini force field for membrane proteins to also allow the simulation of soluble proteins using the Martini and the SIRAH force fields. Moreover, it offers an automated protocol for carrying out the backmapping of the coarse-grained description of the system into an atomistic one.

## 1. Introduction

Cellular processes lean on biological macromolecules and on interactions among them to serve as building blocks for large-scale functional complexes. These often contain many copies of the same or different biomolecules that aggregate through long-range interactions into functional suprastructures. These macromolecular complexes perform their function either in a soluble environment or embedded in the cell membrane. Furthermore, the way in which membrane proteins and their macromolecular complexes associate with and within the lipid bilayer may have an effect on the function of the protein itself. Thus, the availability of techniques that allow a systematic study of these lipids/membrane protein systems spanning large time- and space-scales is fundamental for the understanding of their functions.

Molecular dynamics (MD) simulations have emerged as a powerful tool to study biological systems at varying lengths and timescales. Indeed, all-atom (AA) molecular dynamics simulations are being used by a wide scientific community as a routine to capture biomolecules’ conformational dynamics and local motions. In addition, recent developments in algorithms that reduce the MD sampling [1,2,3,4], and in particular the new advancements in coarse-grained (CG) models, have paved the way to studying the dynamics of macromolecular complexes for timescales up to milliseconds [5]. CG models are a reduced representation of all-atom models that keep the crucial molecular features of the system. This reduced representation of atoms allows not only the simulation of large-scale biological systems, but they also enable faster sampling due to reduced degrees of freedom. As a consequence, CG simulations can cover longer timescales, and therefore provide access to the in-depth knowledge of dynamics of macromolecules, which is beyond the scope of AA simulations [6,7]. Indeed, CG models have been extensively used for studying the protein folding mechanism, structure prediction, protein-membrane systems, and aggregation [8]. For these reasons, the CG protein model development is constantly evolving (see for a review [9]), and their applications are widely used: plenty of studies that have exploited CG force fields, like Martini [10], SIRAH [11], OPEP [12], Shinoda-Devane-Klein (SDK) [13] and ELBA FFs [14], have been published [15], (for a recent review, see [8]).

In 2019, we presented the Martini CoarsE-gRained MembrAne proteIn Dynamics (MERMAID) web server [16] aimed at the preparation, running, and analysis of CG molecular dynamics (CGMD) simulations using the Martini force field tailored to membrane proteins. In this paper, we present a renewed and extended version of our previous server. MERMAID has now evolved into a comprehensive CGMD platform, dedicated to the preparation, running, and analysis of CGMD simulations using the Martini force field version 2.2 for both membrane and soluble proteins. In addition, our platform implements another CGMD force field, namely SIRAH, in an aqueous environment. Finally, we added a backmapping protocol, allowing the users to upload CG Martini structures to be backmapped in the appropriate standalone web server section.

The input can be either an X-ray, NMR, or cryo-EM structure of a protein or models generated by different methods/web servers (for a review on membrane modeling servers, see [17]). In particular, when dealing with models of G-protein coupled receptors (GPCRs) we offer a direct link from our *GOMoDo* web server [18]. The platform funnels the users through several intuitive forms, guiding them, step-by-step, towards the preparation, running, and analysis of a protein CG system. The CGMD Platform implements not only a completely different and more user-friendly interface, but it also includes several novel features: (i) external web servers are no longer required for adding missing atoms within the structure; (ii) a new set of parametrized lipids, as well as different physiological membranes with pre-assembled compositions, are now available. In summary, in this new platform, the users gain the possibility of having a better follow-up of each process due to a constant iterative-mode throughout all the different stages of preparation and running of CGMD simulations. The ‘CGMD Platform’ is freely accessible at https://molsim.sci.univr.it/mermaid/begin.php.

## 2. Results and Discussion

In this section, we describe the different features of the platform, as well as three application cases.

### 2.1. CGMD Platform Architecture

The platform is organized as four different stand-alone web servers. These include: (i) the new version of MERMAID; (ii) the ‘water Martini’ web server dedicated to the simulation of soluble proteins using the Martini force field; (iii) a completely new web server for the preparation and running of CGMD simulations using the SIRAH force field in explicit solvent; (iv) a server dedicated to the backmapping of Martini CG representation of the protein and/or some lipids to an atomistic-level description of the system.

**Figure 1:**
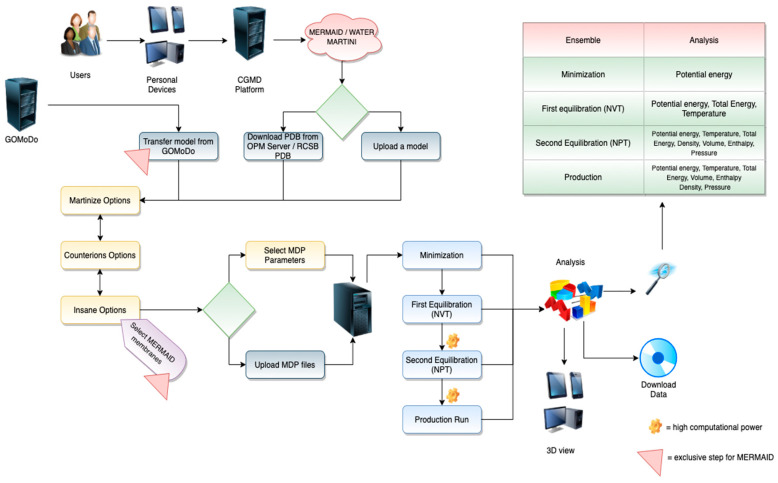
The Martini CoarsE-gRained MembrAne proteIn Dynamics (MERMAID) and Water Martini workflow.

**Figure 2:**
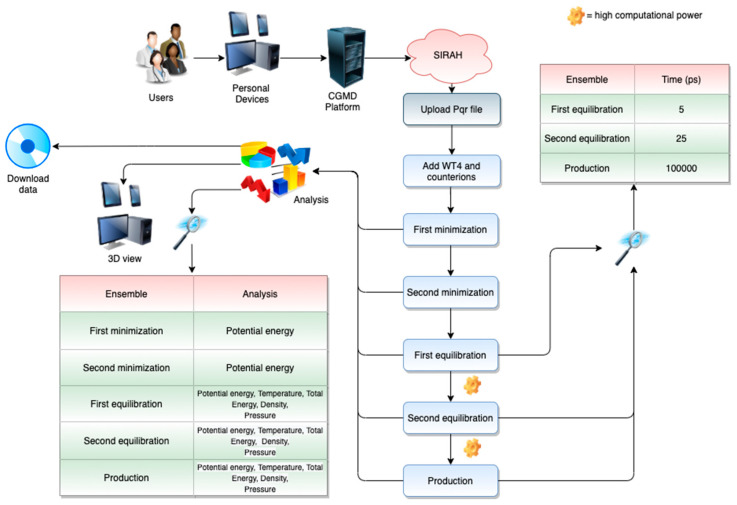
The SIRAH web server workflow.

**Figure 3:**
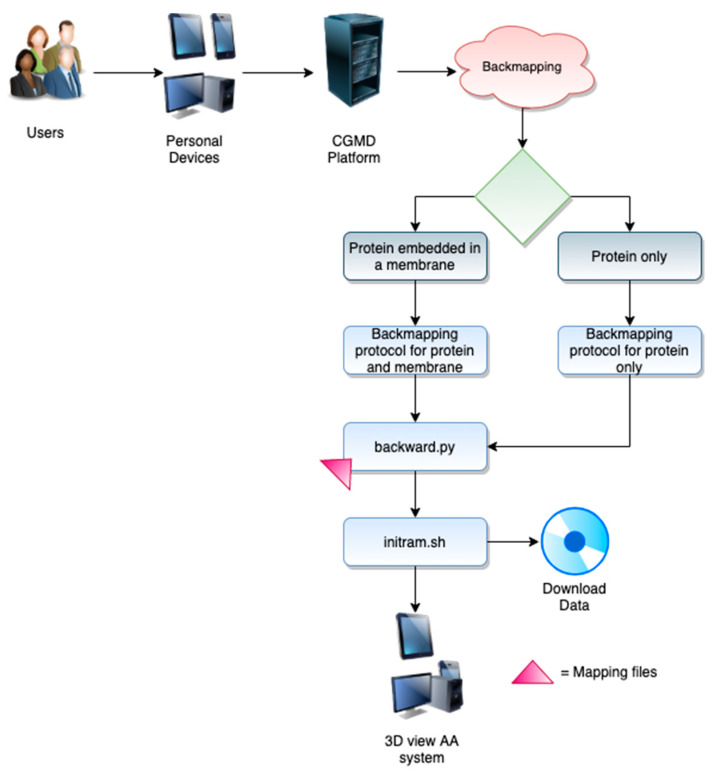
The backmapping workflow.

The workflows for the different web servers can be appreciated in Figure 1, Figure 2 and Figure 3. Each of them can be divided into two different stages, which include the user interface (front-end) and the data retrieval, as well as the back end of the server.

#### 2.1.1. MERMAID and Water Martini Web Servers

MERMAID and Water Martini Web Server workflows are shown in Figure 1.

- **Front-End**
  ○ *File upload*. The users can interact with a renewed web client interface to submit a protein structure. In particular, either a custom PDB structure or a structure automatically downloaded from the OPM Server (https://opm.phar.umich.edu/) [19] (for membrane proteins), or RCSB PDB for soluble proteins, can be submitted (Figure 1). The custom PDB file can be either an experimental or a modeled structure. If the experimental structure is an NMR-derived ensemble, the first conformer is automatically chosen using an in-house script. In the case of GPCRs, the user can employ any modeling program or server (for a review, see [17]). Alternatively, we offer a direct link from our *GOMoDo* web server [18] for the modeling of GPCRs. The models generated using methods other than GOMoDo have to be previously aligned along the *Z*-axis. This can be done manually or by using web servers like the PPM server (https://opm.phar.umich.edu/ppm_server). If the submitted structure contains missing atoms, they will be automatically added using an in-house script that implements the complete_pdb function of Modeller 9.25 [20]. At this point, the user is asked to register in the Modeller webpage (https://salilab.org/modeller/) and to add the corresponding Modeller license key in the appropriate field;
  ○ *Interactive preparation.* This interface allows the users to choose all the parameters for the simulation. The server suggests some default values, but expert users have the freedom to change them according to their needs or directly upload precompiled parameter files. Two versions of the Martini force field are offered, i.e., Martini22 and Elnedyn22. The users are then funneled through different panels. Each panel allows the choice of a large variety of parameters, including those related to the *martinize.py* [21] (Version 2.6) (charges of termini and chain breaks, disulfide bridges, and position restraints) and *insane.py* [22] (PBC type, distance between periodic images, box dimensions, lipid type and abundance in both upper and lower leaflets, etc.) python scripts. Counterions can also be chosen (Na^+^, Ca^2+^, and choline (NC3^+^) as positive ions, Cl^−^ as negative ion) to neutralize the charge of the simulated system. Moreover, expert users can directly upload their own mdp files with custom-made parameters;
  ○ *Membrane selection*. The users can choose from among 57 already parametrized lipids to generate a custom-made membrane composition. On the other hand, the users can select one among several physiological-like membranes, where the known lipidic composition is already set up. The composition of the default membrane offered is the Golgi apparatus membrane [23]. Some of the offered membranes include: (a) Golgi membrane with a composition of: CHOL:18%, POPC:36%, POPE:21%, POSM:6%, POPS:6% and POPI:12%; (b) endoplasmic reticulum membrane: CHOL:8%, POPC:54%, POPE:20% and POPI:11%; (c) plasma membrane: CHOL:34%, POPC:23%, POPE:11%, POSM:17% and POPS:8% and (d) mitochondrial membrane: CDL:22%, POPC:37%, POPE:31% and POPI:6% [23]. Moreover, the user can “model” a customized membrane, varying the concentration and type of lipids. The choice of membrane composition can be done either for both leaflets of the membrane or considering the inner and external leaflets independently. The users have access to all the generated input and output files for each CGMD process at any time. Data can be accessed either from the bookmarked web link or directly from the server user directory by providing the required credentials in the MERMAID search bar, as indicated in the ‘on-the-fly’ documentation;
  ○ *Output and data retrieval*. Results can be viewed and downloaded for 2 weeks (renewal possible) by bookmarking the link, or alternatively by using the corresponding IDs. The full output of the preparation can be downloaded as a compressed archive file including the input, output, and log files of all preparation and simulation steps. The downloaded files can be used to continue the CGMD simulations locally. Experienced users have the possibility of downloading the prepared system and tuning the parameters before running the simulation on their local computer. An array of trajectory analyses is available. These include the calculation of the Root Mean Square Deviation (RMSD), density, pressure, temperature, and gyration radius, among others. The corresponding plots are also visualized. This new version of MERMAID also allows for displaying the simulation run directly on the browser at three different speeds using the NGL Viewer [24].
- **Back-End**. After submitting all files and parameters, the web server creates a local user directory where all operations will be performed. During the initial setup of the system, the atomistic protein structure is converted into a CG representation with the help of the *martinize.py* python script [21]. In the case of membrane proteins, the CG structure is then embedded into a user-defined CG lipid membrane with the help of the python script *insane.py* [22]. Subsequently, the CG simulations are run within our servers. A typical CGMD protocol consists of four phases:
  ○ Minimization run;
  ○ Equilibration runs in two different ensembles, namely canonical ensemble (NVT) and isobaric–isothermal ensemble (NPT);
  ○ Production run continued in an NPT ensemble;
  ○ Analysis of all the trajectories produced during the simulation.

The suggested default parameters are the ones recommended by Martini developers, based on extensive testing [25]: for example, it is advisable to treat coulomb interactions using a reaction-field, as this gives slightly better results at a negligible extra computational cost. A straight cutoff can be used in Martini simulations, with a cutoff distance of 1.1 nm. Good temperature control can be achieved with the velocity rescale (V-rescale) thermostat, using a coupling time constant of at least 0.5 ps. For bilayer systems, the pressure coupling should be semi-isotropic. However, not all systems have been tested and it is recommended that the users perform their own tests.

#### 2.1.2. SIRAH Web Server

SIRAH Web Server workflow is shown in Figure 2.

- **Front-End.** With this web server, the users have the possibility of preparing, running, and analyzing CG simulations using the SIRAH 2.2 force field [26] (Figure 2). This feature allows for using a completely independent approach for running CGMD simulations.
  ○ *File upload*. The users must provide a PDB file containing the correct protonation state for each residue to map at a proper pH the SIRAH CG beads. Since hydrogen nomenclature is not entirely standardized across different software packages, we strongly suggest the use of the pdb2pqr web server [27] (http://server.poissonboltzmann.org/);
  ○ Notice that, if the system contains disulfide bridges, the cysteine residue names involved in disulfide bridges have to be manually edited from “CYS” to “CYX” in the PDB file provided as input. Similarly, to simulate a cysteine in thiolate state, the residue name must be changed from “CYS” to “CYM”. Protonation states of aspartates and glutamates can be set to neutral by editing the residue name to ASH or GLH, respectively;
  ○ *Simulation Parameters*. The server shows the precompiled MD parameters (mdp) files for the simulation. In this case, the parameters are fixed and visible in read-only mode;
  ○ *Running*. After the submission of the file, the user’s job is queued and its status can be monitor at any time during the simulation;
  ○ *Output and data retrieval*. As for the MERMAID web server, the results are stored for 2 weeks. The full output of the preparation can be downloaded as a compressed archive file including the input, output, topologies, and log files of all the preparation and simulation steps. The downloaded files can be used to continue the CGMD simulations locally. The offered analysis tools are the same as for the water Martini case. It can also display the simulation run directly on the browser at three different speeds (1×, 2×, and 5×) using NGL Viewer [24].
- **Back-end.** The protonated model is converted to CG using SIRAH Tools [28] and solvated using pre-stabilized boxes of the WatFour (WT4) CG water model and electrolytes [29]. Each run can be divided into two: solvation and addition of counterions, and the five molecular dynamics steps (two minimization steps, two equilibration steps, and one production step). The mdp files are displayed during the preparation. These parameters were extensively tested and should allow a smooth and fully automated preparation of the system in explicit solvent.

#### 2.1.3. Backmapping Web Server

The implicit loss of resolution of CG representations is a limiting factor when trying to interpret the details of the simulations. Indeed, atomistic level details, such as specific contacts, are the key for understanding molecular recognition and specific intra/intermolecular details. The process of retrieving atomistic details from a CG representation is known as reverse transformation, inverse mapping, or backmapping. There are several different backmapping protocols that follow two different steps, i.e., generation of an atomistic structure based on the CG coordinates, and relaxation step of the generated atomistic structure [30]. Here, we implement the backward program [31] (Figure 3).

- **Front-End**. The backmapping procedure can be reached from an independent menu. The users can backmap a protein in water from the Martini force field to Amber [32], Charmm36 [33] or Gromos [34] force fields. For membrane systems, the following lipids are supported: CHOL, DOPC, DOPE, DOPG, DOPS, DPPC, POPC, POPE, and POPG. Slipids’ force field [35] topologies are used, and consequently the associated protein is backmapped to the amber force field [32];
- **Back-end.** MERMAID backmapping allows reconstructing the protein from a CG to AA representation using the backward program [31]. The latter consists of three scripts and a number of CG to atomistic mapping definition files. For a description of the backmapping procedure, see [31].

### 2.2. Documentation

Extensive documentation for each of the steps and parameters is offered through the use of dynamic pop-ups generated using the Bootstrap Tour tools (https://bootstraptour.com/) locally installed within our machine. The availability of a dynamic, always-accessible guide through the entire process allows the users to be documented “on the fly”, avoiding the opening of new pages or movement along long manual pages.

The tutorial with examples (see Section 2.3) offers pre-calculated systems that can be easily accessed by the user at any time. These cases show all the possible calculations that can be performed through the CGMD platform.

### 2.3. Application Cases

#### 2.3.1. Application Case: The Martini Force Field

As an application case, we present the preparation and running of the *Rhodobacter sphaeroides* translocator protein (RsTSPO), also known as the peripheral benzodiazepine receptor (PDB accession code: 4UC3). TSPO is an 18 KDa membrane protein conserved across the three domains of life [36] with a vast spectrum of roles ranging from an environmental sensor to a functional bioregulator of apoptosis, autophagy, inflammation, along with cholesterol and porphyrin transport [37]. Recently, we presented a CGMD study of this receptor aimed at the characterization of the impact of cholesterol in the formation of multimeric assemblies [38]. In this paper, we claimed that cholesterol affects the size and rigidity of the bacterial translocator proteins to a lesser extent than the mammalian protein. Moreover, an overabundance of cholesterol causes a decrease in the number of contacts at the subunit–subunit interface of the RsTSPO and *Bacillus cereus* TSPO (BcTSPO) systems. For this reason, the role of the sterol could be potentially significant, although the study of these bacterial proteins is not conclusive. Aiming to illustrate the capability of this server, we offer the users the possibility of preparing, running, and analyzing the bacterial TSPO simulation. This application case has also been run, and it is freely accessible here: https://molsim.sci.univr.it/mermaid/public_html/membrane/mrstspo1/run/analysis.php. This system was prepared following the workflow presented in Figure 1 and simulated in a 70% POPG and 30% CHOL model membrane (offered by our CGMD Platform). A total of 305 Na^+^ and 307 Cl^−^ was added to neutralize the system. A 1.5 nm distance between periodic images was applied. The simulation was conducted in four steps (minimization, NVT, NPT, and production) using default parameters. After ~20 ns, the structure was equilibrated, as shown from the RMSD plot. The radius of gyration plot reflects how the protein remained compact during the simulation. Additional plots provided on the analysis page offer tools to assess the behavior of the simulation, which were stable in all the ensembles. Indeed, taking all the analyses together, the user has a system that is prepared to be run in a similar fashion to the one in [38]. During and after the simulation, the users are constantly informed of the status of their calculation through a progress bar positioned at the top of each analysis page, which is always accessible. The backmapping procedure was used at the end of the MD following the steps depicted in the workflow in Figure 3. The final *backmapped.gro* structure is freely accessible in our public Github Repository: https://github.com/JavaScript92/CGMD_Platform. A “Demo Case” button is available from the preparation page to allow an automatic upload of the PDB and running this application case. The reference input file can be downloaded from the same repository.

#### 2.3.2. Application Case: The Water Martini

As an application case, we present the preparation and running of the structure of the orthorhombic form of the hen egg-white lysozyme at 1.5 Å resolution (PDB accession code: 1AKI), which is accessible here: https://molsim.sci.univr.it/mermaid/public_html/water/wusecase/run/analysis.php. This system was prepared following the workflow in Figure 1 and simulated in a 15 nm^3^ cubic box. 305 Na^+^ and 313 Cl^−^ were added to neutralize the system. A 1.5 nm distance between periodic images was applied. The simulation was conducted in four steps (minimization, NVT, NPT, and production) using default parameters. After ~2 ns, the structure was equilibrated, as shown from the RMSD plot. Besides, the expected trend in the radius of gyration plot reflects how the protein remained compact during the simulation. The charts within the analysis page allowed us to assess the stability and convergence of the first 100 ns of simulation. These results indicate that the simulation is ready to begin a production run. For the sake of completeness, to provide an estimation concerning the time gained between an AA and Martini CG simulation carried out in the same system (1AKI) in a water environment, a CG representation, consisting of 29,726 beads, is ~100 times faster than an AA simulation containing 90,273 atoms. Both AA and CG simulations were simulated using the same computational resources (one node and 14 OpenMP threads).

#### 2.3.3. Application Case: The SIRAH Force Field

As an application case of the SIRAH force field [11], we present and run the Binary Complex of Restriction Endonuclease HinP1I with its Cognate DNA (PDB accession code: 2FL3), following the workflow in Figure 2. The results for this demo case can be freely accessed here: https://molsim.sci.univr.it/mermaid/public_html/sirah/s2FL3demo/run/analysis.php. This system was prepared as described in Section 2.1.2 and simulated in explicit solvent. The octahedron box was neutralized adding CG K^+^ and Cl^−^ ions, using the *gmx genion* tool, providing a physiologic ionic strength of 0.15 M. The simulation was performed in five steps: two minimization runs (5000 steps each), two equilibration runs (5 and 25 ps, respectively), and one production run (100 ns). After and during the MD simulation, the users are constantly informed on the status of their simulation through the progress bar positioned at the top of each analysis page, which is always accessible during the running. Within our CGMD Platform, several analyses can be carried out, including 20 dynamic plots (temperature, pressure, potential energy, etc.), a 3D visualization for each MD step, and an “on-the-fly” 3D visualization of the production ensemble. The documentation explains how to retrieve the corresponding jobs. At the end of the simulation, a zip archive containing all the necessary molecular structures (in gro format), topologies, trajectories, and calculated properties (in xvg format) can be downloaded from the analysis page. Finally, the users can run this example by uploading the file 2fl3.pqr available here: https://github.com/JavaScript92/CGMD_Platform.

### 2.4. Usage Statistics

The previous version of the MERMAID web server has been extensively used by different research groups around the world. As of today (31 October 2020), over 100 calculations were performed on our web server, including Covid-19 proteins, ATPases, and several other membrane receptors. For the current version of the CGMD Platform, our group has run several test cases for each of them. For the sake of comprehension, some application cases are reported here: https://molsim.sci.univr.it/mermaid/page/applicationCases.php. Other test files are freely accessible from our Github repository: https://github.com/JavaScript92/CGMD_Platform.

## 3. Materials and Methods

The CGMD Platform web-interface is equipped with HTML 5 (https://www.w3.org/html/), PHP version 7.4.3 (http://www.php.net/), JavaScript (https://www.javascript.com/ and https://www.highcharts.com/), Bash shell (https://www.gnu.org/software/bash/), jQuery 3.4.1 (https://jquery.com/), and Bootstrap 4.4.1 (https://getbootstrap.com/). For the submitted jobs, the web server prepares the input files on our local cluster. All simulations were run in a Linux Ubuntu Server 20.04.1 LTS machine with 12 cores and 32GB of RAM. Results were post-processed (which includes analysis of CGMD trajectory, construction of various data plots, visualization of the production ensemble trajectories) and displayed graphically by using Plotly.js (https://plot.ly).

The current version of CGMD Platform implements: (i) a local version of Gromacs 2019.3 [39] to perform MD simulations and carry out the trajectories analysis as well as the backmapping procedure; (ii) a locally installed version of the Dictionary of Protein Secondary Structure (DSSP) [40] to get the geometrical properties of the secondary structure required for running the Martini force field version 2.2; (iii) all the programs needed for preparing the files for the Martini force field, including the *martinize.py* script [21] for coarse-graining the protein structures, and the *insane.py* script [22] for embedding the protein in the membrane; (iv) the version 2.2 of the SIRAH force field along with SirahTools [28] to prepare a CG version of the solute (downloaded from http://www.sirahff.com); (v) Plotly (http://gnuplot.info/) for data plotting; (vi) NGL viewer [24] to provide an interactive 3D molecular viewer of the molecular dynamics simulation embedded in the web page. Moreover, several Gromacs packages were employed: *gmx energy* tool was used to generate the temperature, pressure, total and potential energy, volume, and enthalpy plots; *gmx density* tool was used to calculate the density of all components of the system; *gmx trjconv* tool was used to convert the trajectories to gro format and remove water as well as lipids from the structures displayed on the browser; *gmx rms* and *gmx gyrate* were applied for the calculation of the RMSD of the protein (the same group was used for the least-squares fit, using the output structure of the NPT equilibration as a reference) and radius of gyration of the protein, respectively.

Finally, since GPCRs share the same topology, the modelled proteins coming from *GOMoDo* are structurally aligned against the Nociceptin receptor in its inactive state, which has been used as a reference structure (RCSB PDB Code: 4EA3, downloaded from the OPM Server), using a locally installed version of LovoAlign [41] to help to obtain a proper orientation in the membrane.

## 4. Conclusions

Understanding the mechanisms and dynamics underlying the function of big macromolecular complexes and membrane proteins embedded in their physiological lipidic environments has always been a challenge for researchers, as the time and space scales needed to gain a comprehensive understanding are very difficult to uncover.

CGMD techniques have been shown to be useful for gaining deeper insights into the mechanisms underlying the function of these systems at time/space-scales that cannot be studied at an atomistic resolution. Due to increasing interest and the availability of more powerful algorithms and hardware, in recent years, several web servers have been developed with the scope to prepare and/or perform different resolution levels of MD simulations, i.e., CHARMM-GUI [42], CABS-flex [43], locPREFMD [44], MDWeb [45], PREFMD [46], ProBLM [47], SMOG [48], UNRES [49], Vienna-PTM [50], MM/CG web server [51]. In particular, the existing web servers provide a web-based graphical user interface to generate various molecular simulation systems and input files to facilitate the usage of common simulation techniques. They are widely used to prepare the input files needed to be run on local clusters, and, in some of them, to run short simulations. Nevertheless, they are not specialized in membrane proteins and they allow neither the selection of different membranes nor the running of CGMD simulations. Our newly developed computational platform implements, together with the previously described features, several novel elements to aid in the preparation and running of CG simulations, such as the availability of a backmapping service, and the possibility of choosing between the Martini or the SIRAH CG force field for carrying out the system preparation. Moreover, the user is constantly guided through the entire procedure by a series of dynamic help pop-ups on each of the pages. This allows more direct and self-aware interaction with the server. Besides, the system offers a set of analyses that can be carried out on the fly to follow the evolution of fundamental features, such as temperature, pressure, and different energy terms during the simulation. Last but not least, it offers the possibility of choosing from among 57 different lipids for custom membrane building, or the availability of ready-to-use realistic models for the most common types of physiological membranes.

Membrane composition and membrane protein oligomerization play a biologically relevant role in cell function. The new platform could prospectively be used for efficiently setting up CGMD simulations of the same protein embedded in different lipidic environments, as well as comparative studies of membrane proteins in different multimeric states, by capitalizing on the low computational cost of CGMD.

To summarize, our novel CGMD Platform offers a user-friendly interface available to all users without any login requirement. The CGMD Platform is the only on-line protocol specifically designed for preparing and running CGMD simulations online: it guides the user step-by-step, therefore enormously reducing the usually time-consuming step of the system-set-up, even for non-expert users. It supports the preparation of complex systems, like membrane protein/lipids, easing the laborious process of membrane assemblies but still allowing them to be completely customizable. In addition, it offers a wide spectrum of highly specialized tools for simulation analyses.
